# Hypoxic stress caused apoptosis of MDBK cells by p53/BCL6-mitochondrial apoptosis pathway

**DOI:** 10.1080/10495398.2023.2299241

**Published:** 2024-01-04

**Authors:** Bin Li, Yangjin Baima, Ji De, Dongxu Wen, Yang Liu, Zhuzha Basang, Nan Jiang

**Affiliations:** a Institute of Animal Husbandry and Veterinary, Tibet Autonomous Regional Academy of Agricultural Sciences, Tibet, China; b Key Laboratory of Animal Genetics and Breeding on Tibetan Plateau, Ministry of Agriculture and Rural Affairs, Tibet, China; c Colleges of Life Science and Technology, Dalian University, Dalian Economic Technological Development Zone, Dalian, China

**Keywords:** Hypoxic stress, apoptosis, MDBK cells, B-cell CLL/lymphoma 6, p53

## Abstract

Hypoxia is an important characteristic of Tibetan plateau environment. It can lead to apoptosis, but the mechanism of apoptosis caused by hypoxic stress needs further clarification. Here, cattle kidney cell MDBK were used as cell model. The effect of hypoxic stress on apoptosis and its molecular mechanism were explored. MDBK cells were treated with hypoxic stress, apoptosis and mitochondrial apoptotic pathway were significantly increased, and the expression of B-cell lymphoma 6 (BCL6) was significantly decreased. Overexpressing or inhibiting BCL6 demonstrated that BCL6 inhibited the apoptosis. And the increase of apoptosis controlled by hypoxic stress was blocked by BCL6 overexpressing. MDBK cells were treated with hypoxic stress, the expression and the nuclear localization of p53 were significantly increased. Overexpressing or inhibiting p53 demonstrated that hypoxic stress suppressed the expression of BCL6 through p53. Together, these results indicated that hypoxic stress induced the apoptosis of MDBK cells, and BCL6 was an important negative factor for this regulation process. In MDBK cells, hypoxic stress suppressed the expression of BCL6 through p53/BCL6-mitochondrial apoptotic pathway. This study enhanced current understanding of the molecular mechanisms underlying the regulation of apoptosis by hypoxic stress in MDBK cells.

## Introduction

Hypoxia is the most important characteristic of Tibetan plateau environment.^1^^,^^2^ In Tibet, hypoxic environment represses the growth and productivity of local animals, including yak.^3–5^ Livestock with high economic performance in plain areas are often introduced into Tibet. But the high-altitude low-oxygen environment leads to the death of a large part of the imported livestock. In recent years, some studies about the effect of high altitude (hypoxic environment) on the cell growth and cell viability have been reported.^6–8^ But the effect of hypoxia on apoptosis and its regulatory mechanism needs further clarification.

At present, there are three classical apoptotic pathways: mitochondrial apoptotic pathway,^9–11^ endoplasmic reticulum emergency pathway^12^^,^^13^ and membrane receptor pathway.^14–16^ In mitochondrial apoptotic pathway, p53 can mediate apoptosis in animals via mitochondrial pathway under environmental stress condition.^17^^,^^18^ Then, p53 protein specifically inhibits the expression of Bcl-2 (Bcl-2 can facilitate the closing of the outer membrane ion channel of mitochondria), but promotes the expression of Bax (Bax promotes the opening of the outer membrane ion channel of mitochondria). When the outer membrane ion channel of mitochondria opens, Cytc is released. Cytc binds to Apaf-1, and then promotes Apaf-1 recruitment and activating Caspase-9. Then the activated Caspase-9 activates Caspase-3, thereby causing apoptosis.^19–22^

In this study, the effect of hypoxic stress-caused apoptosis in MDBK cells and its molecular mechanism was investigated. This study aimed to enhance current understanding of the molecular mechanisms underlying the regulation of apoptosis by hypoxic stress in MDBK.

## Materials and methods

The experimental procedures followed The People’s Republic of China Law on Animal Protection and were approved by the Animal Care Committee of the Tibet Autonomous Regional Academy of Agricultural Sciences.

### The culture and treatment of MDBK cells

MDBK cells were purchased from TONGPAI (Shanghai) Biotechnology CO., LTD (Shanghai, China). Cells were planted in 6-well plates and cultured with Dulbecco’s Modified Eagle’s Medium and Ham’s F12 nutrient medium (DMEM/F12; 12500062, Gibco, CA, USA) containing 10% foetal bovine serum (FBS; 12484028, Gibco), 100 U of penicillin-streptomycin (15070063, Gibco) at 37 °C in a atmosphere of 5% CO_2_ for 12 h.

In the experiment of hypoxia treatment, MDBK cells were randomly divided into two groups, one group was cultured at 37 °C in an atmosphere of 5% CO_2_ (normoxic), the other group was cultured in 5% CO_2_, 95% N_2_ (hypoxia) for 12 h.^23^ In the experiments of genes function, MDBK cells were treated with overexpressed or silenced genes (BCL6 and p53), and then cultured in normal oxygen environment (normoxic) or low-oxygen environment (hypoxia) for 12 h.

### Gene silencing

In this study, BCL6 and p53 were silenced. The specific siRNAs of these genes were synthesized by Sangon Biotech Co., Ltd (Shanghai, China). According to experimental requirements, The siRNAs of BCL6 and p53 were transfected into MDBK cells with Lipo6000™ Transfection Reagent (C0526, Beyotime Biotechnology Co., Ltd, Shanghai, China) according to the protocol.^24^ Briefly, the MDBK cells were plated into six-well plates and cultured with DMEM/F12 nutrient medium containing 10% FBS in normal oxygen environment. When the confluence of cells reached about 80%, the medium was changed with OPTI-MEM medium (22600134, Gibco) and cultured in normal oxygen environment. Then, the specific siRNAs of BCL6, p53 or negative control (100 pmol per well) and the Lipo6000 Transfection Reagent (5 μL per well) were diluted into 125 μL OPTI-MEM medium, respectively, and incubated at room temperature for 5 min. The diluted specific siRNAs and Transfection Reagent were mixed and incubated at room temperature for 15 min. Then, the mixture was added into the OPTI-MEM medium and the cells continued to be cultured for 6 h. The OPTI-MEM medium was changed with DMEM/F12 nutrient medium containing 10% FBS and the cells were cultured for 12 h. The no transfected cells were used as blank control (B group) and the cells transfected with negative control siRNA were used as negative control (NC group). The siRNA sequences used in the current study were shown in Table 1.

**Table 1.** List of siRNA sequences.

Gene name	siRNA sequence (5'-3')	
*BCL6*	Sense	UGUCUUAUGGGCUCUAAACUG
	Antisense	GUUUAGAGCCCAUAAGACAGU
*p53*	Sense	UCUCAUAGCGUUUAAACCCAC
	Antisense	GGGUUUAAACGCUAUGAGAUG
Negative control	Sense	UUCUCCGAACGUGUCACGUTT
	Antisense	ACGUGACACGUUCGGAGAATT

Abbreviations: *BCL6*,B-cell CLL/lymphoma 6.

### Gene overexpression

In this study, BCL6 and p53 were overexpressed. The specific primers of BCL6 and p53 were designed and synthesized by Sangon Biotech Co., Ltd. The sequences of these specific primers were shown in Table 2.

**Table 2.** Primer sequences used for plasmid construction.

Gene name	Primer sequence (5'-3')	
	Forward primer	Reverse primer
*BCL6*	TCCCCCGGGATGGCCTCGCCGGCTGACAGCT (the *Smal* I site is underlined)	GCTCTAGAGCAGGCTTTGGGGAGCTCCGG (the *Xbal* I site is underlined)
*p53*	CGGAATTCATGGAAGAATCACAGGCAGAAC (the *EcoR* I site is underlined)	GAAGATCTGTCTGAGTCAGGTCCCTCTCTCT (the *Bgl* II site is underlined)

Abbreviations: BCL6: B-cell CLL/lymphoma 6.

The total RNA of MDBK cells was extracted with TRIZOL RNA isolation Reagent (15596026, Thermo Fisher, MA, USA) and evaluated using 2100 (Agilent, CA, USA). The cDNA was synthesized with M-MLV reverse transcriptase (2641 A, TaKaRa, Beijing, China). BCL6 and p53 genes were cloned by PCR and the eukaryotic expression plasmids of these genes were constructed according to standard protocol.^25^ The eukaryotic expression plasmid used in this study was pCMV-C-Myc (D2672, Beyotime).

The overexpression plasmids of BCL6, p53 or pCMV-C-Myc were transfected with Lipo 6000 Transfection Reagent. The transfection process was the same as that performed for gene silencing. The amounts of overexpression plasmids and Lipo6000 Transfection Reagent applied were 2.5 μg and 5 μL per well, respectively. The no transfected cells were used as blank control (B group) and the cells transfected with empty vector were used as control (EV group).

### Western blotting analysis

Protein expression of BCL6, p53, β-actin, Cytc, β-Tubulin, VDAC1, Caspase-3 and Caspas-9 was performed by Western blotting (WB). MDBK cells were treated with hypoxic environment, and/or gene overexpression or silencing. The total protein of cells in each group was obtained with cell lysis buffer (P0013, Beyotime Biotechnology Co., Ltd) containing 2% protease and phosphatase inhibitor cocktail (P1050, Beyotime Biotechnology Co., Ltd). The concentration of total protein was tested with BCA protein assay kit (P002, Beyotime Biotechnology Co., Ltd). The WB was completed according to standard protocol.^26^ Briefly, about 30 μg of total protein was separated by 10% sodium dodecyl sulphate-polyacrylamide gel electrophoresis (SDS-PAGE) and then transferred onto a polyvinylidene fluoride (PVDF) membrane (FFP24, Beyotime Biotechnology Co., Ltd). The membrane was blocked with TBSTw (ST671, Beyotime Biotechnology Co., Ltd) containing 5% skim milk powder (SNM436, Biolab Biotechnology Co., Ltd, Beijing, China) at room temperature for 2 h. Then, the membrane was incubated with primary antibody solution (TBSTw containing primary antibody and 5% skim milk powder) at 37 °C for 2 h. The membrane was washed with TBSTw, three times and 5 min per time. Then, the membrane was incubated with HRP-conjugated secondary antibody solution (TBSTw containing HRP-conjugated secondary antibody and 5% skim milk powder) at 37 °C for 1 h. The membrane was washed with TBSTw, three times and 10 min per time and then visualized with Super ECL Plus (P0018S, Beyotime Biotechnology Co., Ltd). Primary antibodies used in this study were as follows: BCL6 antibody (1:500, bsm-60223R, Bioss, Beijing, China); p53 antibody (1:1,000, ab26, Abcam, Cambridgeshire, UK); β-actin antibody (1:500, bsm-33036M, Bioss); Cytc Antibody (1:500, bs-0013R, Bioss); β-Tubulin antibody (1:1,000, ab179511, Abcam), VDAC1 Antibody (1:1,000, ab15895, Abcam); Caspase-3 antibody (1:500, bs-33199M, Bioss); Caspase-9 (1:500, bs-0049R, Bioss). Secondary antibodies were HRP-conjugated. The protein bands were visualized with BeyoECL Plus (P0018, Beyotime).

### Immunofluorescence

The immunofluorescence experiment was carried out according to the conventional method. Cells were cultured on the sterilized cover slips in 6 well plates. When the confluence of cells reached about 50%, cells were then cultured in standard or hypoxic environment. The preparation of samples for immunofluorescence experiment was as previously reported.^27^^,^^28^ Briefly, cells were treated according to the experimental requirements, and then the medium was removed and cells were washed with PBS three times. Then, the cells were fixed with ice-cold methanol at 4 °C for 10 min. Cells were washed with TBSTw (three times, 5 min per time) and blocked with TBSTw containing 5% bovine serum albumin (BSA; ST025, Beyotime Biotechnology Co., Ltd) at room temperature for 1.5 h. Then, cells were incubated with primary antibody solution (TBSTw containing primary antibody and 5% BSA) at 37 °C for 1.5 h. Cells were washed with TBSTw three times (5 min per time) and then incubated with secondary antibody solution (TBSTw containing secondary antibody and 5% BSA) at 37 °C for 1 h in a dark environment. Cells were washed three times with TBSTw (5 min per time) and then incubated with 10 μg/mL propidium iodide (PI; P3566, Gibco) (diluted with PBS) for 10 min at 37 °C in a dark environment. Then, cells were washed three times (10 min per time) with TBSTw. The cells were fixed on the slides with antifade mounting medium (P0126, Beyotime, China) and observed using laser scanning confocal microscopy (LEICA, Germany). Co-localization was analysed with ImageJ2X software. Primary antibody used in this study was p53 antibody (1:1,000, ab26, Abcam, Cambridgeshire, UK) and the secondary antibody used in this study was Goat Anti-Rabbit IgG H&L/AF488 antibody (1:200, bs-0295G-AF488, Bioss).

### Apoptosis analysis

The apoptosis analysis of MDBK cells was evaluated by TiterTACS In Situ Detection Kit-Colorimetric (4822-96-K, Trevigen, MD, USA) according to the manufacturer’s instructions.

### Mitochondrial protein extraction

The MDBK cells were treated with hypoxic environment, and/or gene overexpression or silencing. The treated cells were digested with Trypsin-EDTA Solution (C0201, Beyotime Biotechnology Co., Ltd) and the mitochondria were extracted using Cell Mitochondria Isolation Kit (C0201, Beyotime Biotechnology Co., Ltd) according to the manufacturer’s instructions. VDAC1 and β-tubulin were used as mitochondria and cytoplasmic markers, respectively.

### Statistical analysis

The results were reported as mean ± SD (n = 3). The band of WB and the co-localization of IF were analysed by ImageJ software. Data were analysed using the one-way ANOVA by Excel, differences were considered statistically significant at *p < 0.05*. All data were obtained from at least three independent experiments.

## Results

### Hypoxic stress promotes apoptosis of MDBK cells through mitochondrial apoptotic pathway

**Figure 1.**
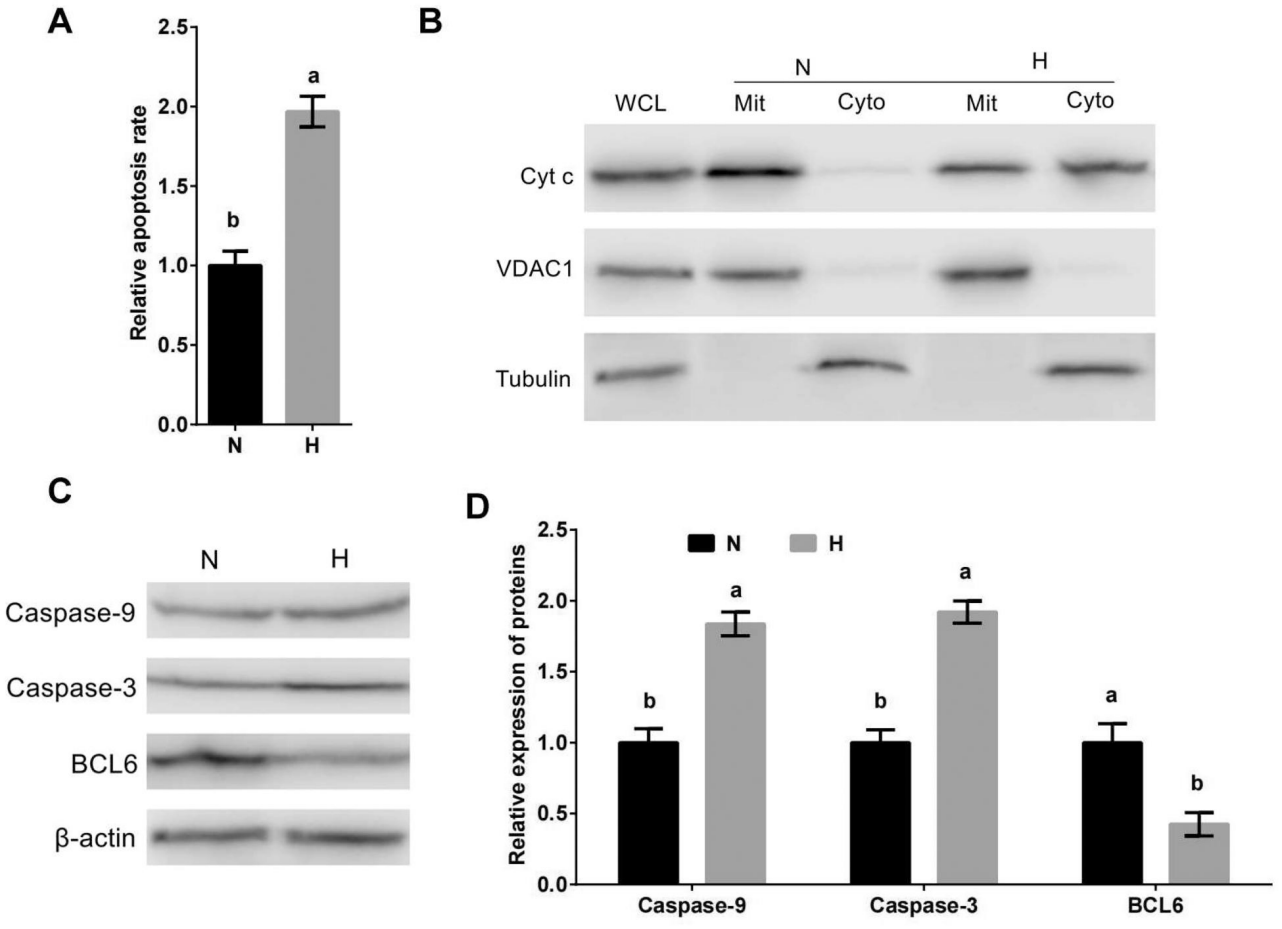
Hypoxic stress promotes apoptosis of MDBK cells through mitochondrial apoptotic pathway. A: Apoptosis rate of MDBK cells under hypoxic environment; B: subcellular localization of Cyt c in MDBK cells treated with hypoxic environment; C–D: the expression of Caspase-9 and Caspase-3 in MDBK cells treated with hypoxic environment. In Fig. 1A, the apoptosis rate of MDBK cells in normal culture group (N) was set to ‘1’. in Fig. 1D, the expression of Caspase-9, Caspase-3 and BCL6 of MDBK cells in normal culture group (N) was set to ‘1’. N: MDBK cells cultured in standard environment; H: MDBK cells cultured in hypoxic environmentin. In the bar charts, different superscript lowercase letters indicated significant differences (*p < 0.05*), while the same letters represent no significant difference (*p > 0.05*).

To investigate if the mitochondrial apoptotic pathway took part in the molecular mechanism of MDBK cell apoptosis caused by hypoxic stress, MDBK cells were cultured in standard or hypoxic environment. The apoptosis rate, subcellular localization of Cyt c, as well as the expression of Caspase-9 and Caspase-3 of MDBK were tested. The result showed that compared with standard cultured group, the apoptosis rate (Fig. 1A), the cytoplasmic localization of Cyt c (Fig. 1B) and the expression of Caspase-9 and Caspase-3 (Fig. 1C–D) of MDBK in hypoxic stress group was significantly increased.

Our previous data suggested that BCL6 may be involved in the regulation of hypoxic stress-mediated apoptosis. The expression of BCL6 in MDBK cells treated with hypoxic stress was tested. The result showed that compared with standard cultured group, the expression of BCL6 (Fig. 1C–D) of MDBK cells in hypoxic stress group was significantly decreased.

Above results suggested that hypoxic stress caused the apoptosis of MDBK, and it worked via mitochondrial apoptotic pathway. And hypoxic stress suppresses the expression of BCL6.

### Hypoxic stress promotes apoptosis of MDBK cells through BCL6

**Figure 2.**
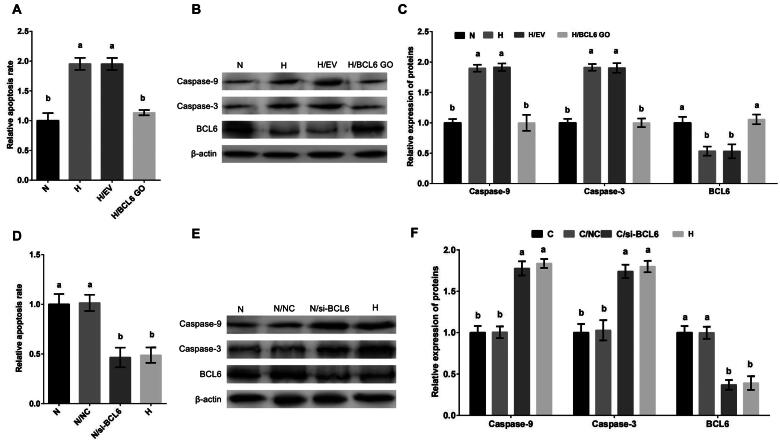
Hypoxic stress promotes apoptosis of MDBK cells through BCL6. A: Apoptotic rate of MDBK cells treated with hypoxic environment or *BCL6* gene overexpression; B-C: the expression of Caspase-9 and Caspase-3 in MDBK cells treated with hypoxic environment or *BCL6* gene overexpression. D: the apoptosis rate of MDBK cells treated with hypoxic environment or *BCL6* gene silencing; E-F: the expression of Caspase-9 and Caspase-3 in MDBK cells treated with hypoxic environment or *BCL6* gene silencing. In Fig. 2A and D, the apoptosis rate of MDBK cells in normal culture group (N) was set to ‘1’. in Fig. 2C and F, the expression of Caspase-9 and Caspase-3 of MDBK cells in normal culture group (N) was set to ‘1’. N: MDBK cells cultured in standard environment; H: MDBK cells cultured in hypoxic environment. EV: cells were transfected with empty vector. NC: cells were transfected with negative control siRNA. BCL6 GO: cells were transfected with *BCL6* gene overexpression vector. si-BCL6: cells were transfected with *BCL6* gene siRNA. In the bar charts, different superscript lowercase letters indicate significant differences (*p < 0.05*), while the same letters represent no significant difference (*p > 0.05*).

To investigate the effect of BCL6 on hypoxic stress-mediated apoptosis, MDBK cells were treated with BCL6 overexpression or silencing, and then cultured in standard or hypoxic environment. The apoptosis of MDBK was tested. The result showed that the apoptosis rate (Fig. 2A), and the Caspase-9 and Caspase-3 expression (Fig. 2B–C) of MDBK treated with hypoxic stress was significantly increased, but these increases were blocked by BCL6 overexpression (Fig. 2A–C). Conversely, the apoptosis rate (Fig. 2D), and the Caspase-9 and Caspase-3 expression (Fig. 2E–F) of MDBK treated with standard cultured was significantly decreased, but these decreases were restored by BCL6 silencing (Fig. 2D–F).

These results suggested that BCL6 is a key negative regulator in the process of hypoxic stress-mediated apoptosis. BCL6 suppressed the apoptosis of MDBK cells after hypoxic stress.

### BCL6 regulated negatively apoptosis of MDBK cells

**Figure 3.**
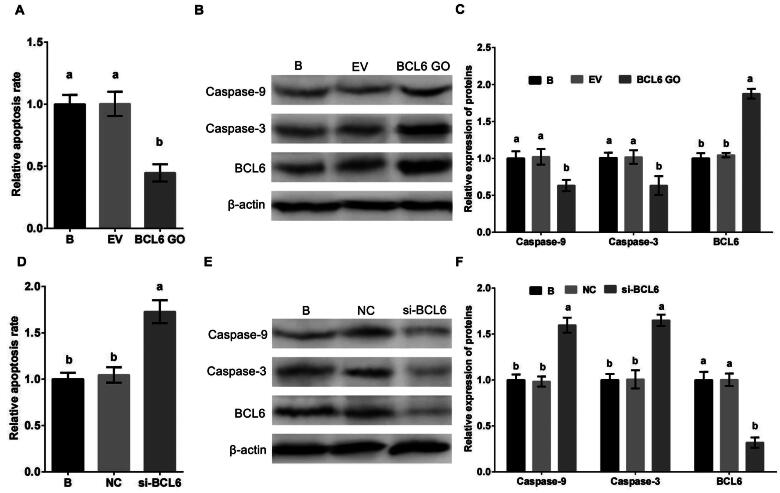
BCL6 negatively regulates apoptosis of MDBK cells. A: the apoptosis rate of MDBK cells treated with *BCL6* gene overexpression; B-C: the expression of Caspase-9 and Caspase-3 in MDBK cells treated with *BCL6* gene overexpression. D: the apoptosis rate of MDBK cells treated with *BCL6* gene silencing; E-F: the expression of Caspase-9 and Caspase-3 in MDBK cells treated with *BCL6* gene silencing. In Fig. 3A and D, the apoptosis rate of MDBK cells with no transfected group (B) was set to ‘1’. in Fig. 3C and 3F, the expression of Caspase-9 and Caspase-3 of MDBK cells with no transfected group (B) was set to ‘1’. B: Cells were no transfected; EV: cells were transfected with empty vector. NC: cells were transfected with negative control siRNA. BCL6 GO: cells were transfected with *BCL6* gene overexpression vector. si-BCL6: cells were transfected with *BCL6* gene siRNA. In the bar charts, different superscript lowercase letters indicate significant differences (*p < 0.05*), while the same letters represent no significant difference (*p > 0.05*).

To investigate the affect of BCL6 on the apoptosis of MDBK cells, cells were treated with BCL6 overexpression or silencing. The apoptosis rate, and the expression of Caspase-9 and Caspase-3 of MDBK cells was tested. The result showed that the apoptosis rate (Fig. 3A), and the expression of Caspase-9 and Caspase-3 (Fig. 3B–C) of MDBK cells were significantly decreased in cells treated with BCL6 overexpression. Conversely, the apoptosis rate (Fig. 3D), and the expression of Caspase-9 and Caspase-3 (Fig. 3E–F) of MDBK cells were significantly increased in cells treated with BCL6 overexpression silencing.

These results suggested that BCL6 was a significant regulator of apoptosis in MDBK cells, it suppresses the apoptosis of MDBK cells.

### Hypoxic stress negatively regulates the expression of BCL6 via p53 in MDBK cells

**Figure 4.**
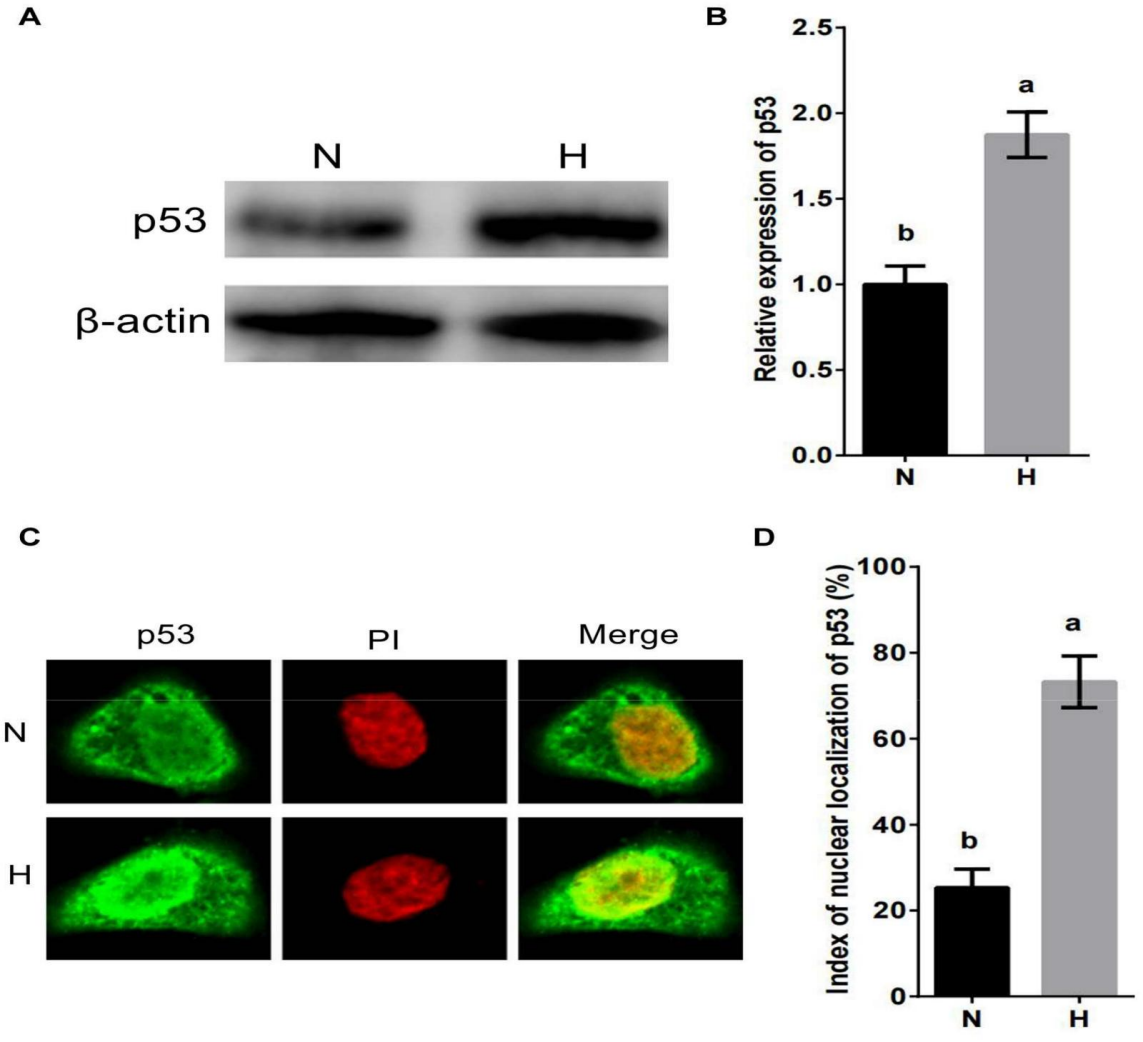
Hypoxic stress promotes the expression and nuclear localization of p53 in MDBK cells. A-B: the expression of p53 in MDBK cells treated with hypoxic environment; C-D: the nuclear localization of p53 in MDBK cells treated with hypoxic environment. In Fig. 4B, the expression of p53 of MDBK cells in normal culture group (N) was set to ‘1’. N: MDBK cells cultured in standard environment; H: MDBK cells cultured in hypoxic environment. In the bar charts, different superscript lowercase letters indicate significant differences (*p < 0.05*), while the same letters represent no significant difference (*p > 0.05*).

p53 is an important factor in hypoxic stress and apoptosis.^29^^,^^30^ To investigate whether the hypoxic stress regulates the expression of BCL6 via p53, MDBK cells were cultured in standard or hypoxic environment. The expression and nuclear localization of p53 were tested. The result showed that the expression of p53 (Fig. 4A–B) and the nuclear localization of p53 (Fig. 4C–D) were significantly increased by hypoxic stress.

**Figure 5.**
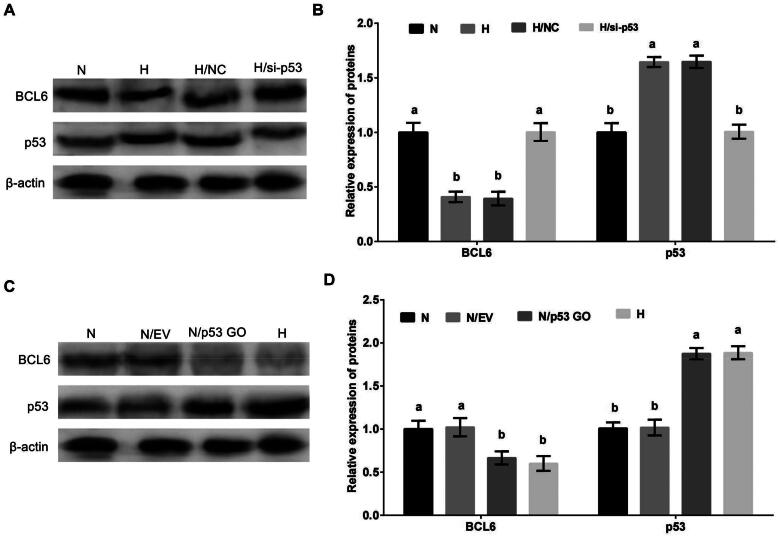
Hypoxic stress negatively regulates the expression of BCL6 via p53 in MDBK cells. A-B: the expression of BCL6 in MDBK cells treated with hypoxic environment or *p53* gene overexpression; C-D: the expression of BCL6 in MDBK cells treated with hypoxic environment or *p53* gene silencing. In Fig. 5B and D, the expression of BCL6 of MDBK cells in normal culture group (N) was set to ‘1’. N: MDBK cells cultured in standard environment; H: MDBK cells cultured in hypoxic environment. EV: cells were transfected with empty vector. NC: cells were transfected with negative control siRNA. p53 GO: cells were transfected with *p53* gene overexpression vector. si-p53: cells were transfected with *p53* gene siRNA. In the bar charts, different superscript lowercase letters indicate significant differences (*p < 0.05*), while the same letters represent no significant difference (*p > 0.05*).

MDBK cells were treated with p53 overexpression or silencing, and then cultured in standard or hypoxic environment. The expression of BCL6 was tested. The result showed that the expression of BCL6 was significantly decreased (Fig. 5A–B) by hypoxic stress, but this decrease was restored by p53 overexpression (Fig. 5A–B). Conversely, compared with hypoxic stress group, the expression of BCL6 was significantly increased by standard culture (Fig. 5C–D), but this increase was blocked by p53 silencing (Fig. 5C–D).

These results suggested that p53 was an important regulator in the regulation of hypoxic stress on the expression of BCL6. Hypoxic stress regulated negatively the expression of BCL6 via p53/BCD6 axis in MDBK cells.

## Discussion

Hypoxia is one of the most important characteristics of living environment of animals in high altitude area of Tibet.^31–33^ In our study, the hypoxic environment was artificially created by control the culture environment of MDBK cells according to previous reports.^34^^,^^35^ We found that the apoptosis was significantly increased in MDBK cells cultured in the artificial hypoxic environment. This result is in line with the expected and it suggests that the artificial hypoxic environment in this study is successful. Importantly, our data demonstrated that the expression of BCL6 of MDBK cells treated with hypoxic environment was significantly decreased, and the mitochondrial apoptotic pathway in MDBK cells treated with hypoxic environment was significantly increased. These results suggested that in MDBK cells, hypoxic environment promote apoptosis through mitochondrial apoptotic pathway and BCL6 may be involved in the regulatory process.

Three pathways of apoptosis are recognized. Mitochondrial apoptosis pathway,^36^^,^^37^ endoplasmic reticulum stress pathway and membrane receptor apoptosis pathway.^38^ In mitochondrial apoptotic pathway, the expression of p53 increased and was activated after the mitochondrial pathway was stimulated. p53 could regulate the expression of BC1-2 and Bax genes. p53 protein can specifically inhibit the expression of BC1-2, but significantly promote the expression of Bax. Bcl-2 and Bcxl bind to VDAC (VDAC is an outer membrane ion channel and also a CYTC releasing channel), and the channel is closed. Bax and Bak can accelerate the opening of VDAC, and after the opening of the channel, Cytc is released. Cytc binds to Apaf-1 upon release, promoting Apaf-1 recruitment and activating Caspase-9 On the other hand, releasing Smac/Diablo, Smac/Diablo binds to IAPs, relieves the inhibition of Caspase-9 activity by IAPS, activates Caspase-9, and activated Caspase-9 activates Caspase-3, thereby causing apoptosis.^39–41^ In our study, we found that hypoxic environment and BCL6 caused apoptosis through mitochondrial apoptotic pathway.

BCL6 is a critical transcriptional repressorl for the development and maintenance of germinal centers (GCs), which are required for generation of an effective humoral immune response.^42^^,^^43^ In this study, we found that BCL6 was involved in the regulation of apoptosis in hypoxic environment and the BCL6 negatively regulated hypoxic stress-mediated apoptosis through mitochondrial apoptotic pathway in MDBK.

The tumour suppressor gene p53 functions in both cell cycle arrest and apoptosis.^29^ Despite considerable advances in understanding as to how p53 regulates growth arrest, the mechanisms by which p53 regulates apoptosis are only just emerging.^44^ In particular, there appears to be a structural and functional separation between the ability of p53 to induce growth arrest and apoptosis.^30^ The tumor suppressor gene p53 plays a major role in the protection of cells from DNA damage.^45^^,^^46^ Activation of the protein in response to irradiation or genotoxic agents, and possibly by other signals, results in growth arrest at the G1 phase of the cell cycle or in apoptosis.^47^ While it has been shown that the ability of p53 to function as a sequence-specific transcriptional activator is necessary for the induction of growth arrest.^48^ Our data indicated that hypoxic environment promoted the expression and nuclear localization of p53. And the overexpression or silencing of p53 found that hypoxic environment inhibited the expression of BCL6 through p53.

## Conclusion

In summary, the current study revealed that in MDBK cells, hypoxia is a environmental stress factor that induces apoptosis. Hypoxia inhibited the expression of BCL6 through p53, and then promoted apoptosis through mitochondrial apoptotic pathway. Our study find that hypoxic stress promotes apoptosis of MDBK cells by p53/BCL6-mitochondrial apoptotic pathway. The findings of this study provide a new insights for understanding the regulation of apoptosis mediated by hypoxic stress in MDBK cells and enhanced our understanding for the mechanisms of hypoxic stress-caused apoptosis.
